# On the Neutrosophic, Pythagorean and Some Other Novel Fuzzy Sets Theories Used in Decision Making: Invitation to Discuss

**DOI:** 10.3390/e23111485

**Published:** 2021-11-10

**Authors:** Pavel Sevastjanov, Ludmila Dymova, Krzysztof Kaczmarek

**Affiliations:** Department of Computer Science, Czestochowa University of Technology, Dabrowskiego 73, 42-201 Czestochowa, Poland; dymowa@icis.pcz.pl (L.D.); krzysztof.kaczmarek@icis.pcz.pl (K.K.)

**Keywords:** novel fuzzy sets theories, intuitionistic fuzzy sets, Dempster–Shafer theory

## Abstract

In this short paper, a critical analysis of the Neutrosophic, Pythagorean and some other novel fuzzy sets theories foundations is provided, taking into account that they actively used for the solution of the decision-making problems. The shortcomings of these theories are exposed. It is stated that the independence hypothesis, which is a cornerstone of the Neutrosophic sets theory, is not in line with common sense and therefore leads to the paradoxical results in the asymptotic limits of this theory. It is shown that the Pythagorean sets theory possesses questionable foundations, the sense of which cannot be explained reasonably. Moreover, this theory does not completely solve the declared problem. Similarly, important methodological problems of other analyzed theories are revealed. To solve the interior problems of the Atanassov’s intuitionistic fuzzy sets and to improve upon them, this being the reason most of the criticized novel sets theories were developed, an alternative approach based on extension of the intuitionistic fuzzy sets in the framework of the Dempster–Shafer theory is proposed. No propositions concerned with the improvement of the Cubic sets theory and Single-Valued Neutrosophic Offset theory were made, as their applicability was shown to be very dubious. In order to stimulate discussion, many statements are deliberately formulated in a hardline form.

## 1. Introduction

During the last 25 years, a number of novel fuzzy sets theories such as Neutrosophic [1,2,3], Pythagorean [4,5], Spherical [6], Picture [7], Cubic [8], etc., with their numerous combinations and modifications were proposed and used mainly in the multiple criteria decision making and the group multiple criteria making but often without previous critical analysis. More recently, articles on these topics were published in journals with relatively low citation indexes, e.g., such as Neutrosophic Sets and Systems or Journal of New Theory, but in recent years, they have increasingly appeared (although in very small numbers) in the reputed scientific journals of the first league such as Applied Soft Computing, Knowledge-Based Systems, Information Sciences, Expert systems with applications, Fuzzy Sets and System, Engineering Applications of Artificial Intelligence, Artificial Intelligence Review, etc. In our opinion, such a caution of editors and reviewers of solid old journals is not caused by their conservatism at all, but by the desire to see, in addition to formal definitions of these theories and numerous theorems, the solution of real methodological and practical problems. In other words, it is desirable to see a fairly simple and understandable explanation of what is really new, and most importantly, useful theories contribute to the solution of uncertainty modeling problems. In this paper, we try to show that these theories besides their undoubted mathematical elegance, very often bring no useful improvements or even desired simplifications in the uncertainty modeling practice. So they should be used (only if their use really needed) with great caution. Besides a critique, we expose the real (in our opinion) problems of these novel theories, which are extensions, in a pure mathematical sense, of the Atanassov’s intuitionistic fuzzy sets (A−IFS). Then, we propose the solution of these problems by redefining the A−IFS in the framework of the more general Dempster–Shafer theory of evidence (DST).

The critical analysis of the novel theories is presented in Section 2. In Section 3, our propositions concerned with the solution of the revealed problems of the novel theories are performed. Section 4 concludes the paper with some remarks.

## 2. Analysis

To alleviate reading this paper, we use a simplified notation whenever possible and avoid some cumbersome formal definitions of the analyzed theories by focusing on their essence.

It was shown in [9] that all the above-mentioned theories and some other ones are the extensions of A−IFS [10], at least in a purely mathematical sense. As we show below, they are not always conceptually justified.

Therefore, we present the basic concepts of A−IFS [10] in the form suitable for our analysis as follows.

### 2.1. Intuitionistic Fuzzy Sets

**Definition** **1.** *An intuitionistic fuzzy set Z in X is a mathematical object*

$$Z=\{<x,\mu_{Z}\left(x\right),\nu_{Z}\left(x\right)>|x\in X\},$$

*where $\mu_{Z}\left(x\right)$ and $\nu_{Z}\left(x\right)$ are the membership and non-membership degrees, respectively, such that*

(1) μ,ν∈[0,1],

(2) 0≤μ+ν≤1.

*Following [10], we call*

(3) π=1−μ−ν

*the hesitation degree.*

It is important that the above definition is not complete. In the literature, there are many papers devoted to the building of the membership and non-membership functions. While analyzing these papers, we have found that their authors do not care of the logically justified properties of these functions in the asymptotic cases when μ(x)=0 or ν(x)=0 or μ(x)=1 or ν(x)=1. In these papers (see, e.g., [11]), we see that according to introduced mathematical presentations of μ(x) and ν(x) and even their graphical illustrations we can observe in *X* the whole areas where, e.g., μ(x)=1, while ν(x)>0 and is even increasing or decreasing in such an area.

We insist that this contradicts common sense. In actuality, if μ(x)=1, then we have a complete (100%) certainty that some event has occurred. Therefore, there is no place for the opposite event and hesitation, i.e., it should be ν(x)=0 and π(x)=0. The similar analyses can easily be conducted for other asymptotic cases.

Consider the more meaningful confirmation of the above statement.

Let us assume that we want to establish the truth of the statement that Admiral Nelson was a gentleman based on the judgments of individuals from a sample of 100 people.

First, consider the case when 20 people called Nelson a gentleman because he bravely fought for his homeland, 30 people did not call him a gentleman because he was a pirate, and the remaining 50 people found it difficult to assess. Then, we can introduce the degree of Nelson’s membership in the “Club of Gentleman” as μ=0.2, the non-membership as ν=0.3, and the hesitation degree as π=0.5.

Then, let us suppose that all people in the sample refused to recognize Admiral Nelson as a gentleman. Obviously, we have μ=0. It is clear that in this case we have ν=1 and π=0, since we simply do not have people who could say anything else. Indeed, since everyone completely rejected Nelson’s belonging to gentlemen, then there are physically no those who would have hesitated in the assessment; hence, π=0. At last, the complete rejection of Nelson’s belonging to gentlemen (μ=0) means the complete acceptance of the opposite hypothesis of Nelson not being a gentleman, i.e., ν=1. This can be inferred formally as follows: since by definition, it should always be μ+ν+π=1, and in our case, we have μ=π=0, then ν=1.

The similar analyses can easily be performed for other cases.

Therefore, the above definition should be complemented by the asymptotic conditions:

(4) If μ=0, then ν=1, π=0; ifμ=1, then ν=0, π=0.

There are no special suggestions concerning dependencies between the components μ,ν and π in the A−IFS, but they are mutually dependent by default.

There are some methodological problems of the A−IFS and A−IVIFS discussed in the literature.

The first of them is the difficulty an expert experiences when assigning real values to μ and ν. It was noted that sometimes experts insist on such values that their sum μ + ν happened to be greater than 1, which is unacceptable in the framework of the intuitionistic theory.

To avoid or at least alleviate this problem, some extensions (corrections, modifications) of the A−IFS were developed.

The chronologically first of them is the so-called Neutrosophic Set theory (NST) [1,2,3,9].

### 2.2. Neutrosophic Set Theory

In terms of A−IFS, the basics of Neutrosophic Set theory (NST) [3] can be presented as follows:

**Definition** **2.** *A Neutrosophic Set Z in X is a mathematical object*

$$Z=\{<x,\mu_{Z}\left(x\right),\nu_{Z}\left(x\right),\pi_{Z}\left(x\right)>|x\in X\},$$

*where $\mu_{Z}\left(x\right)$, $\nu_{Z}\left(x\right)$ and $\pi_{Z}\left(x\right)$ are the membership, non-membership and hesitation degrees, respectively, such that*

(5) μ,ν,π∈[0,1],

(6) 0≤μ+ν+π≤3forthecompletelyindependentcomponents,

(7) 0≤μ+ν+π≤1forthedependentcomponents.

It is easy to see that the conceptual difference between the NST and the A−IFS is the introduction of the hypothesis of complete independence of the components. It is stated in [9] that this is true to all the novel theories considered. Unfortunately, this fact is not usually taken into account in the works, where NST was applied. We say “unfortunately” since we have deep doubts about the validity of this hypothesis of the components mutual independence from its practical applicability point of view; of course, this does not concern its mathematical correctness.

According to the independence hypothesis, the event μ=1,ν=1 and π=1 is allowed in the NST and in this case, the constraint (6) is fulfilled. Suppose μ,ν and π are the degrees of truth, false and indeterminacy, respectively (this is the notation used in the NST). Thus, if we deal with a complete truth (μ=1), then in compliance with the formal logic and common sense, the measure of false is 0 (ν = 0) without any indeterminacy (π = 0). The similar reasoning is valid for the cases of complete false and complete indeterminacy.

Therefore, we can say, e.g., that the high degree of truth is obligatory accompanied by the low degrees of false and indeterminacy. Since a lot of similar statements can be formulated and all of them are in line with common sense and the formal logic, we can conclude that the independence hypothesis, i.e., the independence of components μ,ν and π is not valid at all in the framework of the problem at hand; by contrast, we deal with the mutually dependent components.

It is interesting that the events μ=1,ν=1, π=1 and μ=0,ν=0, π=0 are interpreted in [9] as a paradox, and its definition is treated as a merit of the NST. In our opinion, generally, it seems to be more reliable to use theories, which have no paradoxes, e.g., the A−IFS theory, in our case. It can be seen that these paradoxes take place in the two opposite asymptotic limits μ=1,ν=1, π=1 and μ=0,ν=0, π=0 of the NST. The theories with such controversial asymptotic properties cannot be recommended to be used.

However, it does not mean that the results that are interesting from a mathematical point of view cannot be obtained using the NST formalism [12].

In the case of mutually dependent components, the main constraint 0≤μ+ν+π≤1 in the NST seems to be more fruitful than that in the A−IFS (π+μ+ν=1). This was quickly discovered and the so-called Picture fuzzy sets theory (PFS) was proposed.

### 2.3. Picture Fuzzy Sets Theory (PFS)

In the framework of the PFS [7], the so-called refusal degree *r* such that μ+ν+π+r=1 was introduced.

According to the semantic of the word “refusal”, it means the rejection of something. Thus, based on common sense, we can say that in our case, it should mean the rejection from the problem consideration. In other words, if the refusal degree is greater than 0, then we should not begin studies at all, and if we only begin to analyze a problem, then the refusal disappears. Thus, the introduction of the refusal degree seems to be senseless even from a semantics point of view.

The operational laws defined on NST objects were introduced in [13]. In [14], it was shown that they suffer from a set of important drawbacks. Therefore, in [14], the simplified operational laws were proposed, but their properties were not studied. The operational laws proposed in [13,14] are the direct mechanical extension of those defined earlier in the A−IFS theory. In our paper [15], we showed six revealed important drawbacks of these operations, which should undoubtedly be inherited in operations with NST objects. In summary, we can say that the NST probably is interesting from a purely mathematical point of view but seems to be unpractical and therefore a senseless extension of the A−IFS based on illogical premises.

### 2.4. Pythagorean Fuzzy Set Theory

Consider the Pythagorean fuzzy set theory (PFS) introduced in [4]. Its definition differs from that of A−IFS by only one assumption: instead of constraint (2), the following one $\mu^{2}+\nu^{2}<1$ is proposed. The definition of the PFS is based on the simple reasoning [5], which here we present in some modified form as follows:

Suppose we have the membership degree μ=0.8 and the accompanied degree of non-membership ν=0.5. Then, μ+ν>1, and we have no an intuitionistic fuzzy number, but if in this example we replace μ and ν with $\mu^{2}$ and $\nu^{2}$, respectively, we obtain $\mu^{2}+\nu^{2}<1$. Thus, according to [5], in the last case, we deal with the truly intuitionistic fuzzy number.

At first glance, this reasoning is attractive, but some questions arise: If μ is a membership degree, then what is the sense of $\mu^{2}$ described in a natural language? Why it was chosen the squared degree if the use of the third, fourth, and so forth degree looks even more promising?

Since we have not found answers to the above questions, we suppose that they are generic methodological problems of the Pythagorean fuzzy set theory.

One more practical question is as follows: Does the Pythagorean fuzzy set theory always provide $\mu^{2}+\nu^{2}<1$? The answer is negative.

Let μ=0.9 and ν=0.5; then, μ+ν>1 and $\mu^{2}+\nu^{2}>1$, too.

To be fair, we note that the author of the theory claims only a very limited application: “...we observe that intuitionistic membership grades are all points under the line x+y≤1 and the Pythagorean membership grades are all points with $x^{2}+y^{2}\leq 1$. We see then that the Pythagorean membership grades allow for the representation on a larger body of non-standard membership grades than intuitionistic membership grades” [5].

We agree with this statement if the reasonable answers on the above questions are found.

Moreover, if the Pythagorean fuzzy set theory is an extension of the A−IFS, then it extends only the possibility of violating the basic constraint μ+ν≤1. Thus, the practical usefulness of such an extension seems to be at least very discussable.

### 2.5. Spherical Fuzzy Sets

The so-called Spherical fuzzy sets [6] may be treated as an extension of the Pythagorean fuzzy sets as they are based on the assumption that $\mu^{2}+\nu^{2}+\pi^{2}\leq 1$. It is easy to see that such an extension possesses all the negative (doubtful) properties of the Pythagorean fuzzy sets and additionally complicates the problem (see more in [9]).

The common problem of both of the last theories based on the squared degree and A−IFS as well is the choice of appropriate values of the components μ,ν and π, such that the constraint ≤1 has to be met under objectively existing but not strictly defined (practically unknown) dependence between the components.

It is easy to imagine how difficult this practical problem really is for experts involved in the solution of, e.g., a decision-making task.

The method for the solution of this and some other collateral problems is presented in the next section.

Consider the foundations of the Cubic sets theory.

#### Cubic Sets

We should start directly from the formal definition since we have not found in the literature the real worthy-of-attention facts or examples that can serve as cornerstones of the theory foundations.

In [8], the following definition was first introduced:

**Definition** **3.** *Let X be a non-empty set. By a cubic set in X, we mean a structure*

$$A=\{\langle x,A(x),\lambda (x)\rangle ,x\in X\},$$

*where A is an interval valued fuzzy set in X and λ is a fuzzy set in X.*

Let us try to provide some reasonable practical interpretation of this abstract definition. Suppose in the decision-making problem, we have a set of local criteria $x_{1},x_{2},\ldots ,x_{n}$ and two experts. The first expert prefers to use interval valued fuzzy assessments $A(x_{i})$ of alternatives relative to criteria, and the second expert prefers real-valued fuzzy estimations $\lambda (x_{i})$. Then, for some given alternative, we can introduce the Cubic set *A*=$\left\{\langle x_{i},A\left(x_{i}\right),\lambda \left(x_{i}\right)\rangle ,x_{i}\in X\right\}$. It is easy to see that in the considered example, we deal with the typical group decision-making task, which can be successfully solved using well-known methods. There is no need for the artificial theoretical superstructure in the form of the Cubic set. Moreover, it undesirably complicates the problem as the adaptation of the Cubic set to the specificity of the group decision-making task leads to the occurrence of new unnecessary mathematical objects and often cannot be provided without additional restrictions. In our opinion, it is impossible to imagine a practical problem, where the Cubic set is really useful. Meanwhile, the attractiveness of the Cubic sets for mathematicians is coursed by the unlimited possibility to build new mathematical objects and theories. Suppose in the above example we involve a new expert, who prefers intuitionistic fuzzy estimates, to say θ(x). Then, we can create a new, so to say, a Super Cubic set SCS= $\left\{\langle x,A(x),\lambda (x),\theta (x)\rangle ,x\in X\right\}$ and so on.

In our opinion, the most daring theory was proposed in [16]. This theory allows negative and greater than 1 values of membership degrees. There are some basic definitions introduced in [16], but here, we analyze only the most general one:

**Definition** **4.** *For T(x),I(x) and F(x) being the degrees of truth, indeterminacy and false, respectively, a Single-Valued Neutrosophic Offset A is defined as follows:*

$$A=\{\left(x,\langle T(x),I(x),F(x)\rangle \right),x\in U\},$$

*such that there exist some elements in A that have at least one neutrosophic component that is >1 and at least another neutrosophic component that is <0.*

For example: *A* = $\left\{\left(x_{1},\langle 1.3,0.3,0.2\rangle \right)\right\}$, $\left\{\left(x_{2},\langle 0.1,0.4,-0.8\rangle \right)\right\}$, since $T(x_{1})$ = 1.3 >1 and $F(x_{2})$ = −0.8 <0.

There is a crucial example in [16], which clarifies the author’s reasoning that we critically analyze: “In a given company a full-time employer works 40 h per week. Let’s consider the last week period. Helen worked part-time, only 30 h, and the other 10 h she was absent without payment; hence, her membership degree was 30/40 = 0.75 <1. John worked full-time, 40 h, so he had the membership degree 40/40 = 1, with respect to this company. But George worked overtime 5 h, so his membership degree was (40+5)/40 = 45/40 = 1.125 >1. Thus, we need to make distinction between employees who work overtime, and those who work full-time or part-time. That’s why we need to associate a degree of membership strictly greater than 1 to the overtime workers.”

The crucial drawback of this reasoning is the lack of the clear definition of fuzzy classes, which memberships are estimated. We can see here two distinct fuzzy classes: the class of employees working at least no more than 40 h a week and the class of employees that works more than 40 h. The first class is presented by the membership function rising from 0 to 1 in the interval [0,40] of worked hours and equal to 0 if the sum of worked hours is greater than 40. The second class is defined by the membership function increasing from 0 to 1 in the interval of worked hours from 40 to $H_{max}$, where $H_{max}$ is the maximal allowed by government (and trade unions) value of worked hours. We can see that such an obvious reasoning does not allow membership degrees greater than 1. The incorrect reasoning of the author of [16] is also based on the implicit mechanical conjunction of two different classes with not intersected supports. Of course, such a conjunction can be made, but the resulting fuzzy class and the corresponding membership function should have a new sense reflecting a synthetic nature of a new class. In the considered case, we can introduce the class of “hard working employees” with the membership function rising from 0 to 1 in the interval $\left[0,H_{max}\right]$.

Let us turn to the example: “Yet, Richard, who was also hired as a full-time, not only did not come to work last week at all (0 worked hours), but he produced, by accidentally starting a devastating fire, much damage to the company, which was estimated at a value half of his salary (i.e., as he would have gotten for working 20 h that week). Therefore, his membership degree has to be less that Jane’s (since Jane produced no damage). Whence, Richard’s degree of membership, with respect to this company, was −20/40 = −0.50 <0.”

As we are analyzing only the last week, we can see that Richard does not belong to any of the classes described above. It is a member of a practically unlimited class of those who do not work for a given company. We can significantly narrow this class by considering only those people who, by their actions or inaction, cause damage to the company (the most harmful are the top managers of competing firms). This way we can estimate the maximum damage $D_{max}$ (it does not matter in money or equivalent worked hours), which can be inflicted on the company by an external detractor. Thus, the class of external (non-working for the company ) people who bring company damages can be presented by the membership function varying from 0 to 1 in the interval of damages [0,$D_{max}$]. There is no place for any negative membership degree. In our opinion, in this case, the author of [16] mistakenly tries to mechanically combine completely different fuzzy classes without introducing any of their even informal definitions. There are some other more simple examples in [16], but they all suffer from the above-analyzed incorrect reasoning.

Finally, we can say that the theory introduced in [16] allows negative and greater than 1 values of the degree of membership is based on the incorrect premises and reasoning.

Summarizing this section, we can say that there is an important feature common for all analyzed novel theories (generalizing intuitionistic fuzzy sets and other theories): they were developed contrary to the ancient Occam’s wisdom: “Entities should not be multiplied unnecessarily.”

It is not a good practice only to criticize without making any propositions. Thus, in the next short section, we briefly present an approach that makes it possible to eliminate most of real problems of the A−IFS theory extending it in the framework of the Dempster–Shafer theory of evidence.

## 3. Propositions

First, we should say that an approach announced above, which eliminates most of real problems of the A−IFS theory, we have already developed earlier in [17] and used for the solution of multiple criteria decision-making problems. Therefore, here we present it only in contest of the problems discussed in the previous section.

For the readers convenience, we present here some basic definitions of the Dempster–Shafer theory of evidence (DST) [18,19] in the extent needed for our analysis.

A Dempster–Shafer (DS) belief structure is represented by a basic probability assignment (bpa) which is a mapping *m* from subsets of *X* into a unit interval, $m:2^{X}\to \left[0,1\right]$ such that $m\left(\emptyset \right)=0,\sum_{A\subseteq X}m(A)=1$. The subsets of *X* with positive (non-zero) values of *m* are called focal elements.

The measure of belief is defined as

(8) $$Bel\left(Y\right)=\sum_{\emptyset \neq Z\subseteq Y}m(Z).$$

The measure of plausibility can be presented as

(9) $$Pl\left(Y\right)=\sum_{Z\cap Y\neq \emptyset }m(Z).$$

It is seen that Bel(Y)≤Pl(Y).

A measure of ignorance about an event *Y* and its complementary $\bar{Y}$ is treated as the length of the so-called belief interval [Bel(Y),Pl(Y)] (BI). It can also be considered as an imprecision of the “true probability” of *Y* [19].

A cornerstone of DST is the problem of combination of the evidence with different origins.

This problem is very important, and many different combination rules may be found in the literature. The corresponding reviews are presented in [20,21].

It was shown in [17] that for x∈X, (*Z* is a fuzzy class in *X*) the triplet $\mu_{Z}\left(x\right)$, $\nu_{Z}\left(x\right)$, $\pi_{Z}\left(x\right)$ can be treated as the basic probability assignment in the framework.

The reasons behind this are as follows:

It is easy to see that if we analyze some situation in the context of *A*-IFS, we should implicitly consider the following hypotheses: Yes: x∈Z, No: x∉Z, (Yes,No): no of the hypotheses x∈Z and x∉Z cannot be excluded (the hesitation takes place).

Thus, $\mu_{Z}\left(x\right)$ can be considered as the probability or evidence of x∈Z, i.e., as the focal element of the basic assignment function: m(Yes) = $\mu_{Z}\left(x\right)$. Then, in this way, we assume m(No) = $\nu_{Z}\left(x\right)$. Because $\pi_{Z}\left(x\right)$ is usually interpreted as the degree of hesitation, the obvious proposition is m(Yes,No) = $\pi_{Z}\left(x\right)$. Since by definition $\mu_{Z}\left(x\right)+\nu_{Z}\left(x\right)+\pi_{Z}\left(x\right)=1$, we can conclude that $\mu_{Z}\left(x\right)$, $\nu_{Z}\left(x\right)$ and $\pi_{Z}\left(x\right)$ represent a regular basic assignment function in the DST.

Then, in the framework of the DST, we obtain the belief and plausibility measures as follows:

$$Bel_{Z}\left(x\right)=m\left(Yes\right)=\mu_{Z}\left(x\right)\;and\;Pl_{Z}\left(x\right)=m\left(Yes\right)+m\left(Yes,No\right)=\nu_{Z}\left(x\right)+\pi_{Z}\left(x\right)=1-\nu_{Z}\left(x\right).$$

As the result, the following definition was introduced in [17] (its shortened version is presented here):

**Definition** **5.** *An intuitionistic fuzzy set Z in X is a mathematical object*

(10) $$Z=\{<x,BI_{Z}\left(x\right)>|x\in X\},$$

*where $BI_{Z}\left(x\right)=\left[Bel_{Z}\left(x\right),Pl_{Z}\left(x\right)\right]$ is the belief interval, $Bel_{Z}\left(x\right)=\mu_{Z}\left(x\right)$ and $Pl_{Z}\left(x\right)=1-\nu_{Z}\left(x\right)$ are the measures of belief and plausibility that x∈X belongs to the set Z⊆X.*

Thus, the basic components of the A−IFS are redefined in the framework of the DST.

There is a well-known general scientific paradigm that if a problem cannot be solved within the framework of a given theory, then it is advisable to look for a solution using a more general theory, if there is one.

Thus, first, we need to make sure that the DST can really be considered as a generalization of the A−IFS.

It can be seen that only a few of the interior elements of the DST were used above to redefine the whole set of the A−IFS components. Therefore, the DST seems to be a more general (generic) theory in relation to A−IFS, and we can expect that by operating with the A−IFS objects in the semantics of the DST, we can obtain new results that are not available in the A−IFS and solve its unsolved problems.

This is not surprising since the DST acts as a generalizing theory for a number of other well-known theories that model uncertainties. It is known that the probability and possibility theories are in some sense the asymptotic cases of the DST; in [15,22], we have shown that the DST is the useful generalization of the A−IFS, as well as of its interval-valued extension; in [23], we have proved that the same is true in relation to the hesitant and interval-valued hesitant fuzzy sets; in [24], we have introduced the DST extension of the rule-base evidential reasoning in the intuitionistic fuzzy setting, which was used in [25] for the type 2 diabetes diagnostic; in [26], we have used the DST extension of the A−IFS in the generalization of the TOPSIS method. There is such a strong link between rough set theory and the DST that it is difficult to find a more general theory among them [27]. Some arguments in favor of the DST may be found in [28], but we have no doubts that the DST is more general, because in contrast to the rough set theory, it generates many derived theories.

Let us consider the advantages that bring us the DST extension of the A−IFS.

An important problem of the conventional A−IFS is that it is often hard for a decision maker or expert to assign such values to μ and ν, such that in all cases, the inequality μ+ν≤1 is held.

In the framework of the DST extension, we deal with the belief interval BI = [Bel,Pl], not directly with the μ and ν, although we remember that initially for the belief and plausibility measures, we had Bel=μ and Pl=1−ν=μ+π.

Thus, a decision maker is proposed to assign to Bel and Pl, the values from the interval [0,1]. If a decision maker assigned some value to Bel he or she should obligatorily assign a greater value to the plausibility measure. This follows both from the definitions of Bel=μ and Pl=μ+π and from the semantics of a natural language.

For example, suppose we have some event *x*. Then, Bel(x) is our belief in *x*, and Pl is the plausibility of *x* or the degree, in which we cannot exclude *x*. It is clear that in these circumstances, a decision maker has no choice but to use such degrees Bel and Pl that the inequality Bel≤Pl has always been performed. This is quite natural due to the semantics of a natural language and common sense and therefore does not cause any difficulties for a decision maker. The inequality Bel≤Pl by definition is equivalent to μ≤1−ν and therefore to μ+ν≤1.

Thus, we can see that the considered problem of the conventional A−IFS does not exist at all in the framework of the DST extension. It is also important that as a consequence, there is no need for such somewhat artificial and heuristic theories as the Neutrosophic, Pythagorean and Spherical sets and their derivatives.

Here, we present the additional important practical argument in favor of the separate use of our approach based on the DST extension (see Definition 5 above) without any terms from A−IFS. In communications with experts, we found that they easily and willingly set the values of the degree of membership. Very often in technological practice, particular criteria are formulated in the form of trapezoidal fuzzy numbers, to which the top is the interval of the best values of the parameter and the bottom is the interval of acceptable values. Experts are used to this and are willing to use it. However, with the degree of non-membership, difficulties arise.

Let us say the expert sets μ = 0.2. Then, he or she automatically sets ν = 0.8. Moreover, he or she considers other values of ν as erroneous. It is quite possible that such a “probabilistic” style of thinking, which is a consequence of a good higher education, prevents the widespread use of methods of the A−IFS to solve real-world problems, although there are other reasons of a rather psychological nature. However, in any case, this is the problem of the A−IFS.

At the same time, when the experts were asked to evaluate the BI = [Bel,Pl], there were no noticeable problems. It is characteristic that sometimes the experts offered their own interpretations of the belief interval BI. Among them the most successful and transparent was the interpretation of the BI=[Bel,Pl] as the interval between pessimistic and optimistic assessments of the membership degree.

It is worth noting that our approach is flexible enough. For example, if someone wants to only use the μ and ν formalism of the A−IFS, he or she can first obtain from experts the estimations of BIs (avoiding the above-discussed problems concerned with the estimation of ν ) and then convert BIs into μ and ν based on the expressions μ = Bel, ν = 1−Pl.

We should note here that in practice, we can meet the data available with such a structure that the μ and ν appear objectively without expert’s judgments (see examples in [24,29]).

Summarizing, we can state that the introduced DST redefinition of the A−IFS is fruitful and justified from both theoretical and practical points of view.

An important problem of the conventional A−IFS is the drawbacks of operational laws defined on intuitionistic fuzzy values (IFV). In [15], using convincing examples, we showed that the conventional operations with IFS have six undesirable properties (two of them were noted earlier in [30]), which may provide unacceptable results when they are used for the solution of real-world problems (see [15] for the detailed analysis).

No wonder, the contradictions are seen even in definitions of these operations, e.g., multiplication is used in the definition of the sum operation and the power operation is applied to define the multiplication by a scalar.

Therefore, based on the dual nature of a belief interval which can be treated as an interval enclosing a true power of statement (argument) that x∈X belongs to the set A⊆X and as a usual regular interval, in the framework of the DST extended A−IFS, in [15], we have inferred the operational laws defined on the belief intervals representing the corresponding IFVs without above-mentioned six revealed drawbacks. The last statement was justified formally using relevant theorems. The DST extension of the interval-valued A−IFS is presented in [22].

Therefore, we can say that introduced extension of the A−IFS is valuable and justified from both theoretical and practical points of view.

Unfortunately, we have no propositions concerned with the improvement of the Cubic sets theory and the Single-Valued Neutrosophic Offset as we cannot imagine a practical problem, where these theories can be really useful.

## 4. Conclusions

Foundations of such novel theories such as Neutrosophic, Pythagorean, Spherical, Picture, Cubic and some other sets are analyzed from the points of view of their logical consistency, applicability and common sense compliance. The drawbacks of these theories are revealed and discussed. It is shown that the cornerstone hypothesis of the Neutrosophic sets theory, i.e., the assumption of independent components does not conform to common sense and leads to the senseless paradox results in the asymptotic cases of this theory. It is shown that the Pythagorean sets theory is based on the premises, which cannot be rationally clarified. It is also an important drawback that this theory does not completely eliminate the declared problems, for which the solution the theory was developed. The important methodological problems of other analyzed theories were revealed and discussed. Many of the novel theories, such as Neutrosophic, Pythagorean, Spherical and Picture sets theories, were developed to solve interior problems of the Atanassov’s intuitionistic fuzzy sets. Since the results obtained within the framework of these criticized novel theories appeared to be significantly worse than expected, in this paper, an alternative approach based on the extension of the intuitionistic fuzzy sets in the framework of the Dempster–Shafer theory is proposed. It is shown that this approach makes it possible to effectively solve the problems of the Atanassov’s intuitionistic fuzzy sets theory.

The practical applicability of the Cubic sets theory is considered as very dubious. Of course, it can be successfully used in the purely mathematical studies, but in the decision-making tasks, it cannot provide new results unobtainable by the known simpler methods. We understand that such a conclusion is a debatable one, but the aim of our paper, as reflected in its title, is to initiate a discussion.

The membership degrees greater than 1 and less then 0 in the Single-Valued Neutrosophic Offset theory are shown to be only the results of an incorrect reasoning based on the unjustified mechanical conjunction of completely different fuzzy classes with disjoint supports.

The main future direction of our studies is the implementation of the methodology described in Section 2 in the solution of the real-world problems such as the development of automatic trading systems on the currency market and the raw material sources selection, as well as the solution of additional theoretical problems arising at the implementation stage.

## Data Availability

Not applicable.
